# Research of CO_2_ and N_2_ Adsorption Behavior in K-Illite Slit Pores by GCMC Method

**DOI:** 10.1038/srep37579

**Published:** 2016-11-29

**Authors:** Guohui Chen, Shuangfang Lu, Junfang Zhang, Qingzhong Xue, Tongcheng Han, Haitao Xue, Shansi Tian, Jinbu Li, Chenxi Xu, Marina Pervukhina, Ben Clennell

**Affiliations:** 1 Research Institute of Unconventional Petroleum and Renewable Energy (RIUP&RE), China University of Petroleum (East China), Qingdao 266580, Shandong, PR China; 2 School of Geosciences, China University of Petroleum (East China), Qingdao 266580, Shandong, PR China; 3 CSIRO Energy Flagship, 26 Dick Perry Ave, WA 6151, Australia; 4 Shaanxi Province key laboratory of lacustrine shale gas accumulation and development, xi’an 710000, Shaanxi, PR China; 5 State Key Laboratory of Heavy Oil Processing, China University of Petroleum, Qingdao 266580, Shandong, PR China; 6 College of Science and Key Laboratory of New Energy Physics & Materials Science in Universities of Shandong, China University of Petroleum, Qingdao 266580, Shandong, PR China

## Abstract

Understanding the adsorption mechanisms of CO_2_ and N_2_ in illite, one of the main components of clay in shale, is important to improve the precision of the shale gas exploration and development. We investigated the adsorption mechanisms of CO_2_ and N_2_ in K-illite with varying pore sizes at the temperature of 333, 363 and 393 K over a broad range of pressures up to 30 MPa using the grand canonical Monte Carlo (GCMC) simulation method. The simulation system is proved to be reasonable and suitable through the discussion of the impact of cation dynamics and pore wall thickness. The simulation results of the excess adsorption amount, expressed per unit surface area of illite, is in general consistency with published experimental results. It is found that the sorption potential overlaps in micropores, leading to a decreasing excess adsorption amount with the increase of pore size at low pressure, and a reverse trend at high pressure. The excess adsorption amount increases with increasing pressure to a maximum and then decreases with further increase in the pressure, and the decreasing amount is found to increase with the increasing pore size. For pores with size greater larger than 2 nm, the overlap effect disappears.

Because of the continuous growing of the global energy demands and the insufficient conventional oil and gas resources, more and more attentions are being focused on the unconventional energy resources^1^. The great success of both exploration and development in North America has proved the shale gas to be the most promising unconventional energy recently^2^. Shale gas consists of both free gas within inter granular pores or natural fractures and adsorbed gas on or underneath the surface of insoluble organic matter (kerogen) or inorganic minerals^3^. Previous studies have confirmed that the amount of adsorbed gas constitutes about 20–85% of the total gas in place of shale gas reservoirs^4^. Therefore, the behavior of adsorbed gas plays an important role in both exploration and development of shale gas. Whereas the hydrocarbon gases account for large proportion of shale gas, the contents of CO_2_ and N_2_ and their impact on shale gas adsorption behavior are non-negligible^5^. The content of CO_2_ can be as high as 10% for some wells in New Albany Shale, and the content of N_2_ sums up to 65% for some wells in Antrim Shale^5^. Together with the fact that illite is one of the most important adsorbent components in shale^6^,^7^, the investigation of the mechanisms of CO_2_ and N_2_ adsorption behavior in illite is valuable for both exploration and development of shale gas. Furthermore, the investigation will also provide the theoretical basis for the enhancement of shale gas recovery by injecting CO_2_ and the storage of CO_2_ in shale.

While the adsorption capacity of shale has been widely studied experimentally within a limited temperature and pressure range^8^,^9^, the experiments can only help to estimate the excess adsorption amount without distinguishing the mechanisms of adsorption behavior. Molecular simulation methods, on the other hand, have been recently introduced to investigate the gas adsorption mechanisms in both kerogen and clay over large pressure and temperature ranges^10^,^11^,^12^,^13^,^14^,^15^,^16^. However, there is still some insufficiency in the molecular simulation investigation which needs to be further studied. First of all, recent molecular simulation studies on the shale gas adsorption behavior in clays were mainly focused on Na-montmorillonite structure and the gas of CH_4_ and CO_2_^11^,^14^,^17^. Other clays, such as illite and kaolinite, also play an important role in shale gas adsorption behavior^6^,^7^, and other gas, such as N_2_, also exists in shale^5^. Secondly, the suitability of simulation system was usually not discussed before the simulations in the previous studies. Thirdly, the expected bulk gas density, which will be used when calculating the excess adsorption amount, was commonly obtained by the equation of state rather than the simulations^11^,^12^,^18^. However, the difference in bulk gas density obtained by these two methods will induce uncertainties to the excess adsorption amount. Fourthly, the excess adsorption amount was conventionally expressed per unit mass of adsorbent^15^,^18^ to compare with experimental measurements in previous studies. But to the best of our knowledge, the comparability between these two results (simulation and experiment) has to be further discussed. Finally, the mechanisms of the overlap effect of adsorption behavior in micropores and its corresponding phenomenon on isotherms have not been discussed systematically.

To solve these problems, the grand canonical Monte Carlo (GCMC) simulations on CO_2_ and N_2_ in K-illite with varying pore sizes are performed at the temperature of 333, 363 and 393 K over a range of pressures up to 30 MPa. Before the investigation of adsorption mechanisms, the impact of cation dynamics in the simulation system and the pore wall thickness of the simulation cell is discussed to prove the suitability of the simulation system. For the purpose of calculating the excess adsorption amount, the GCMC simulations in empty simulation box are performed to get the expected bulk gas density. The expression unit of excess adsorption amount is discussed, and then the comparisons between the molecular simulation results and the experimental measurements are made to confirm the validity of the simulations. The overlap effect of the adsorption behavior is also discussed in detail by comparing the gas density profiles, the gas-clay interaction energies and the excess adsorption isotherms for pores with varying sizes, and the mechanisms of overlap effect in micropores are finally revealed.

## Methods

### Models

**Figure 1:**
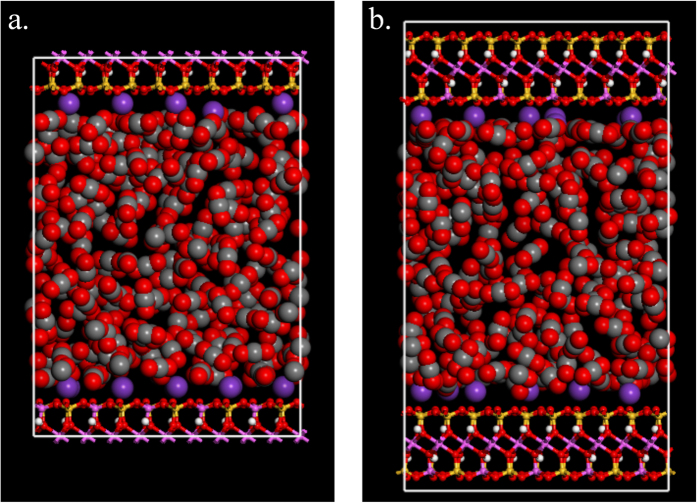
A snapshot of (**a**) type I and (**b**) type II simulation cells with carbon dioxide molecules in between. Carbon dioxide molecules and potassium cations are represented by spheres and clay layers are represented by ball-stick structures, with the color scheme: O, red; H, white; Si, yellow; Al, pink; K, blue; C, grey. (For interpretation of the references to color in this figure legend, the reader is referred to the web version of this article).

The adsorption system consists of both adsorbent (K-illite) and adsorbate molecules (CO_2_ or N_2_). The K-illite is represented by pyrophyllite-1Tc^19^,^20^ with the unit cell formula of K_x_[Si_(8-x)_Al_x_](Al_4_)O_20_ (OH)_4_^21^. In each unit cell one silicon (Si) is substituted by one aluminum (Al) in the tetrahedral sheet (*x* = 1), causing of −1.0 e charge per unit cell. The negative charge caused by the substitution is balanced by the interlayer potassium cations (K^+^)^22^. To investigate the effect of the pore wall thickness on adsorption behavior, two types of clay simulation cells (types I and II) are built (as shown in Fig. 1). Type I simulation cell is a patch with two half layers of the clay sheet, and with the interlayer slit-like pore space in between (as shown in Fig. 1a). Type II simulation cell is a patch with two complete layers of the clay sheet, and also with the interlayer slit-like pore space in between (as shown in Fig. 1b). Counter cations and gas molecules are presented in the slit pore. Three-dimensional periodic boundary condition is applied to both type I and type II simulation cells, which are composed of 15 (5 × 3 × 1) unit cells and 30 (5 × 3 × 2) unit cells respectively, making up 2.58 nm in x-dimension by 2.69 nm in y-dimension for both type. In order to investigate the influence of pore size (defined as the distance between the planes of the centers of oxygen atoms in the inner surface of the tetrahedron) and the pore wall thickness on adsorption behavior, 4 pore sizes of 0.74 nm, 1 nm, 2 nm and 3 nm, respectively are selected for type I simulation cells and 2 pore size of 0.74 nm and 3 nm, respectively for the type II. In the simulations, the cutoff distance is 1.25 nm, so the simulation cell is repeated in z-dimension if its size is less than 2.5 nm to make sure it is not smaller than 2 times of the cutoff distance. The CLAYFF force field is used for clay structure^23^, by which the clay sheets are considered to be rigid, and no bending potential is considered for clay sheets.

The adsorbate molecules, including CO_2_ and N_2_, are simulated using transferable potentials for phase equilibria (TraPPE model)^24^. For CO_2_ molecule, the C-C bond length and O-C-O bond angle are fixed at the experimental value of 1.16 Å and 180°, respectively. Point charges of +0.7 e and −0.35 e are placed on carbon site and oxygen site, respectively. N_2_ molecule consists of three sites. Each nitrogen atom is modeled by a Lennard-Jones site separated by the experimental bond length of 1.10 Å. The gas-phase quadrupole moment of N_2_ (Q = −1.4 × 10^−26^ esu) is reproduced by placing point charges of −0.482 e on each Lennard-Jones site. To maintain charge neutrality, a point charge of +0.964 e is placed at the mass center of the N_2_ molecule.

In the clay-gas system, the adsorption behavior is governed by the combination of gas-surface and gas-gas interactions, which are dominated by both van der Waals forces and coulomb forces^12^,^14^. The van der Waals interactions between pseudoatoms, which belong to different molecules, are described by pairwise-additive Lennard-Jones 12–6 potentials^25^. Cross interactions between unlike atoms are calculated by the Lorentz-Berthelot combining rules^26^,^27^. The Coulomb energy of the interaction is inversely proportional to the distance of separation *r*_*ij*_^23^.

### Simulation details

In this study, GCMC simulations are performed to investigate the adsorption behavior of CO_2_ and N_2_ in K-illite with varying pore sizes, temperatures and pressures using the Sorption model of Material Studio and RASPA 2.0^28^,^29^. In the GCMC simulations, the chemical potential of the gas (*μ*), the volume (*V*), and the temperature (*T*) of the system are fixed. The chemical potential (*μ*) relates to reservoir gas or bulk gas pressure (*P*) by 
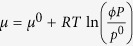
, where *p*^*0*^ and *μ*^*0*^ are the standard pressure and chemical potential, respectively, and *ϕ* is the fugacity coefficient obtained from the NIST thermodynamic properties^30^. The GCMC simulations are firstly performed on both type I and type II simulation cells at the temperature of 363 K with the pore size of 0.74 nm and 3 nm to investigate the influence of the pore wall thickness. We then perform the GCMC simulations on type I simulation cells to reveal the adsorption mechanisms at the temperature of 363 with varying pore sizes from 0.74 nm to 3 nm, and at the varying temperature of 333, 363 and 393 K with the pore size of 2 nm. The GCMC simulations with both rigid and dynamic potassium cations are performed on Type I simulation cell with 2 nm pore size using RASPA 2.0 to investigate the impact of the cation dynamics. The temperature of the simulation system is 363 K (90 °C), and the pressure is fixed to be 1 MPa, 10 MPa and 30 MPa, respectively. To obtain the bulk density of CO_2_ and N_2_ at each temperature and pressure, the GCMC simulations are performed in an empty box of 3 × 3 × 3 nm in *x*-, *y*-, *z*-dimension, respectively. Each GCMC simulation run consists of 1 × 10^6^ steps for equilibration and 1 × 10^6^ steps for ensemble average.

## Results And Discussion

### The suitability of the simulations

The comparability of characteristics between the simulation system and natural illite mineral is a key factor that influences the validity of simulations. Thus, it is necessary to investigate the suitability of the simulation system at the beginning of the GCMC simulations. As the cation dynamics and the pore wall thickness are the critical factors that influence the suitability of simulation system, we discuss the impact of these two factors before the mechanism investigation.

#### The impact of cation dynamics

In the adsorption experiment, the sample is usually pretreated by simultaneously heating to over 100 °C and vacuuming to eliminate the water and impurity gas^8^,^31^, after which the potassium cations will locate at the surface of the illite sample. The gas is then injected into the sample cell and the gas molecules will adsorb on the pore wall surface. If the interaction between the gas molecules and the pore wall surface is much stronger than that between the potassium cations and pore wall surface, the potassium cations located on the pore wall surface will be replaced by gas molecules. In this case, the GCMC simulations with rigid potassium cations will not be able to reflect exactly the adsorption behavior in natural illite mineral.

**Figure 2:**
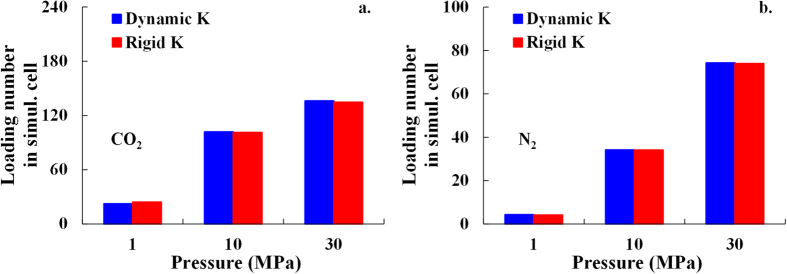
The loading number of (**a**) CO_2_ and (**b**) N_2_ molecules in simulation cell with both rigid and dynamic K cations.

The loading numbers of CO_2_ and N_2_ with both rigid and dynamic potassium cations are given in Fig. 2, which shows that the differences in the loading number of both CO_2_ and N_2_ between rigid and dynamic potassium cations are slight and negligible. It can be inferred that the potassium cations located on the illite surface can’t be replaced by gas molecules. This is because the interaction between potassium cations and illite surface is stronger than that between the gas molecules (CO_2_ and N_2_) and the illite surface. As a result, both rigid and dynamic potassium cations in the simulation cells are suitable to investigate the adsorption behavior of CO_2_ and N_2_. To simplify the simulation system, the rest of the simulations in this paper is performed only with rigid potassium cations using Material Studio.

#### The impact of the pore wall thickness

As shown in Fig. 1, in both type I and II simulation cells, the *x*- and *y*- dimension and the slit pore size are the same. However, the clay mass, which is determined by the number of clay sheet layers (pore wall thickness), is different. Specific surface area is a property of solids defined as the total available surface area per unit mass of the adsorbent. For the simulation cell with a specific *x*- and *y*- dimension and the slit pore size, the specific surface area depends mainly on the clay mass, which is determined by the pore wall thickness. In other words, the specific surface area of clay can reflect the slit pore wall thickness.

For the simulation cell with the counter cations in its pore space, the available surface is built up by rolling a probe atom with a certain dynamic radius over the inner clay surface and cation surface, also known as the “Connolly surface” when the probe is spherical^32^,^33^, and then the total surface area can be obtained. The specific surface area of the simulation cell can be computed by dividing the mass of the simulation cell from the total available surface area, which is generally expressed in the unit of m^2^/g. The specific surface areas for the simulation cells together with the kinetic diameters of the adsorbate gas molecules are listed in Table 1. The specific surface areas of the simulation cells are 1038.92 (CO_2_) and 1015.76 (N_2_) m^2^/g for type I, and 522.40 (CO_2_) and 517.04 (N_2_) m^2^/g for type II. The specific surface area of natural illite mineral varies in different publications, from 2.26 m^2^/g analyzed by nitrogen^7^ to 64 m^2^/g analyzed by atomic force microscopy^34^. It is obvious that the specific surface areas of the simulation cells are significantly larger than that of the natural illite mineral. This is because the slit pore wall thickness of the simulation cell is markedly thinner than that of the natural illite mineral.

**Figure 3:**
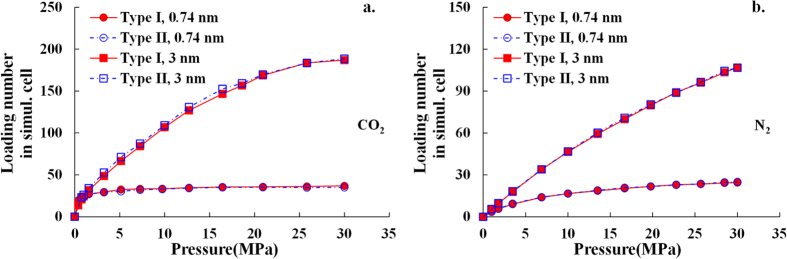
Comparison of the total loading number of (**a**) CO_2_ and (**b**) N_2_ in both type I and II simulation cells at the temperature of 363 K (90 °C).

In a simulation cell with more layers of clay sheet (thicker pore wall), the additional clay sheet within the cutoff distance may also interact with the gas molecules in pore space, which in turn may affect the adsorption behavior. In order to investigate the suitability of the simulation cells, the influence of pore wall thickness on adsorption behavior should be studied. The GCMC simulations are performed on both type I and II simulation cells with the pore size of 0.74 nm and 3 nm at the temperature of 363 K (90 °C). Simulation cell with thicker pore wall is not investigated since the separation between extra clay sheet and gas molecules in pore space would be out of the cutoff distance, and thus the interaction is negligible. The comparison of the total loading number of adsorbate gas molecules in type I and II simulation cells with the pore size of 0.74 nm and 3 nm is shown in Fig. 3. It can be seen that for a given pore size, the difference in the total loading number of both CO_2_ and N_2_ molecules between in type I and type II simulation cells is negligible.

Therefore, the pore wall with thickness greater than half layer of clay sheet (type I simulation cell) will not affect the adsorption behavior anymore. We can conclude that, even though the thickness of the simulation cells is much thinner than that of natural illite mineral, it is suitable to use both type I and II simulation cells to investigate the adsorption behavior for both CO_2_ and N_2_. Considering the calculation cost, all the simulations mentioned in the rest of the paper are performed on type I simulation cells with less atoms.

### Gas distribution in pore space

Having proved the suitability of the simulation system, we firstly investigated the gas distribution in pores with varying sizes.

**Figure 4:**
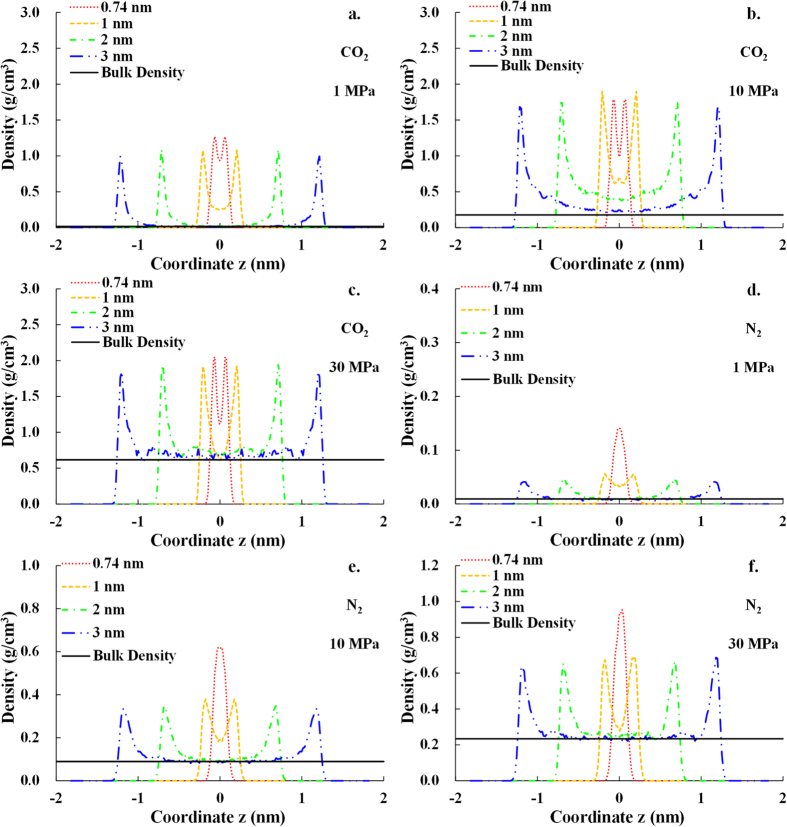
The density profile of both CO_2_ and N_2_ at different pressures with varying pore sizes along z direction which is perpendicular to the clay surface. (**a**) CO_2_ at 1 MPa; (**b**) CO_2_ at 10 MPa; (**c**) CO_2_ at 30 MPa; (**d**) N_2_ at 1 MPa; (**e**) N_2_ at 10 MPa; (**f**) N_2_ at 30 MPa.

The gas density profiles along *z* direction (perpendicular to the clay surface) of CO_2_ and N_2_ at the temperature of 363 K (90 °C) are shown in Fig. 4. For the pore sizes of 1 nm, 2 nm and 3 nm, gas molecules close to the slit pore wall interact strongly with the clay surface, forming two distinct density peaks as the strong adsorption layers (the typical strong and weak adsorption layer are marked in Fig. 4f). Gas molecules out of the strong adsorption layers also interact weakly with the clay surface, forming the small density peaks as the weak adsorption layers, whose density is much lower than that of the strong adsorption layers but higher than the expected bulk phase density (as shown in Fig. 4). If the gas molecules in the center of slit pore space are far enough from both side of the clay surface and the gas-clay interaction is negligible, the gas density would fluctuate around or almost equal to the expected bulk phase density.

As the pore size decreases, the sorption potential from both side of the pore wall surface overlaps, so the gas molecules in the center of the slit pore space begin to interact with both side of the clay surface. As a result, the gas density of adsorption layers overlaps^14^,^35^,^36^. Because the smaller pore size causes stronger overlap effect, the gas density of the weak adsorption layers increases as the pore size decreases. When the pore size drops to 0.74 nm, there is not enough space for the weak adsorption layers, and therefore the strong adsorption layers begin to overlap. When the space is not big enough to form two separated strong adsorption layers, a single distinct density peak occurs (as shown in Fig. 4d–f). We can deduce that if the pore size continues to decrease, there would be not enough pore space to form the single density peak, and thus the density peak would diminish and finally disappear.

As shown in Fig. 4, the gas density of CO_2_ is obviously higher than that of N_2_, indicating stronger interaction between CO_2_ and clay than that between N_2_ and clay. For both CO_2_ and N_2_, the difference between the gas densities of the strong adsorption layers for different pore sizes is not significant, except for the pore size of 0.74 nm, for which the strong adsorption layers overlap. The weak adsorption layers overlap at the pore size of 1 nm and the strong adsorption layers overlap at the pore size of 0.74 nm at all pressures shown in Fig. 4. Consequently, both the strong adsorption layers and the overlapped weak adsorption layers contribute to the adsorption amount for the pore size of 1 nm, and the overlapped strong adsorption layers contribute for the pore size of 0.74 nm. For the pore size of 2 nm and 3 nm, the contributors to the adsorption amount vary with the pressure. At low pressures, 1 MPa for CO_2_ and 1 MPa and 10 MPa for N_2_, only strong adsorption layers with no weak adsorption layers contribute to the adsorption amount, because almost no weak adsorption layer occurs. At high pressures of 10 MPa and 30 MPa for CO_2_ and 30 MPa for N_2_, the weak adsorption layers occur, so both the (overlapped) weak adsorption layers and strong adsorption layers contribute to the adsorption amount. We can conclude that the difference in the adsorption amount between different pore sizes (from 1 nm to 3 nm) mainly depends on the (overlapped) weak adsorption layers and the relevant volumes, because the densities of strong adsorption layers are similar.

It should be pointed out that the interlayer potassium cations play dual roles on the adsorption behavior. One is that the potassium cations with positive charge interact with the point charges in gas molecules. The other one is that the potassium cations compete with gas molecules to occupy the adsorption site on clay surface. Under the dual effect of potassium cations, the density of the strong adsorption layers for the pore size of 0.74 nm is slightly lower than that of larger pore size at the pressure of 10 MPa for CO_2_ (as shown in Fig. 4b).

### Interaction energy

We then investigated the affinity between gas and clay surface in pores with different sizes by comparing their interaction energies.

**Figure 5:**
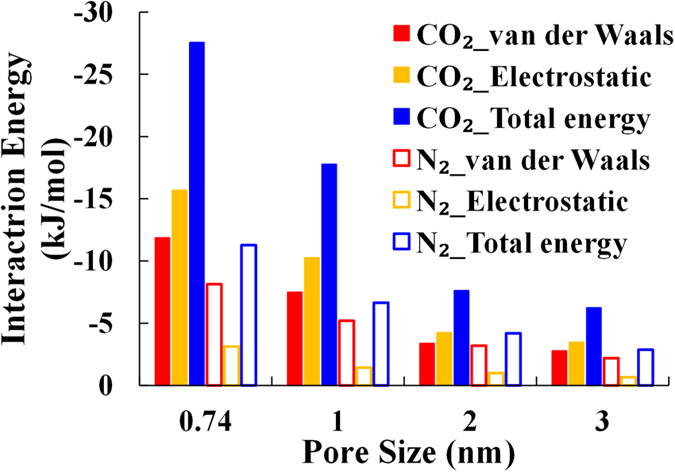
The interaction energy between gas molecules (CO_2_ and N and clay with varying pore sizes.

The interaction energy between gas and clay surface is analyzed with varying pore sizes at the pressure of 10 MPa (as shown in Fig. 5). The interaction energy, expressed by kJ per mole of gas, is obtained by averaging the gas-clay interaction energy per mole of simulation cell by the number of gas molecules uptake in the simulation cell.

The interaction between gas molecules (CO_2_ and N_2_) and the clay surface is mainly caused by van der Waals force and Coulomb force. The polarizability of CO_2_ (29.1 × 10^−25^ cm^3^/molecule) is larger than that of N_2_ (17.4 × 10^−25^ cm^3^/molecule)^37^, causing stronger van der Waals interaction between CO_2_ and clay (including K cations). The molecular electric quadrupole moment of CO_2_ (4.3 × 10^−26^ esu cm^2^)^38^ is almost 3 times as large as that of N_2_ (1.5  × 10^−26^ esu cm^2^)^39^, making stronger Coulomb interaction between CO_2_ and clay (including K cations). Consequently, the total CO_2_-clay interaction energy is much larger than total N_2_-clay interaction energy as shown in Fig. 5.

For both CO_2_ and N_2_, the gas-clay interaction energy increases as the pore size decreases, especially when the pore size is smaller than 2 nm (as shown in Fig. 5). This is because the sorption potential from both side of pore wall surface overlaps when the pore size is smaller than 2 nm^12^,^14^,^35^,^36^,^40^, which makes the interaction between gas and clay surface stronger. The overlap of the sorption potential in micropores makes the gas density higher in the smaller pore size, this is consistent with the gas density profile shown in Fig. 5.

### Excess adsorption

#### The calculation and expression

The output of GCMC simulation is the loading number of adsorbate gas molecules at a certain pressure and temperature, rather than the adsorption amount. Excess adsorption amount in GCMC simulation is defined as the additional loading amount of adsorbate gas compared with the gas amount in the same volume in absence of pore walls at the same pressure and temperature. Subtraction of the latter from the former can thus yield the excess adsorption amount. The number of moles of excess adsorption, *n*_*ex*_, is defined as


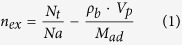


where *N*_*t*_ is the total loading number of adsorbate gas molecules in the pore space, *Na* is the Avogadro constant of 6.02 × 10^23^ mol^−1^, and *M*_*ad*_ (g/mol) is the molar mass of the adsorbed gas molecules. The bulk gas density *ρ*_*b*_ (g/cm^3^) is calculated using the GCMC simulations in the 3 × 3 × 3 nm empty box. The free pore volume *V*_*p*_ (cm^3^) is limited by the “Connolly surface”^32^,^33^.

In order to compare the simulation results with the experimental measurements and other published simulation results, the excess adsorption amount should be expressed in a proper unit. Even though different adsorption mechanisms like pore filling and single/multiple layer adsorption exhibit in different pore sizes^41^, the adsorption behavior is generally believed to be dominantly caused by the gas-mineral surface interaction. Therefore, the adsorption capacity of the clay with a given pore size distribution mainly depends on the total available surface area.

As discussed previously, the specific surface area of the simulation cell is significantly larger than that of natural illite mineral. As a consequence, the total surface area as well as the adsorption capacity per mass of simulation cell is markedly larger than that of natural illite. In order to compare the simulation results with the experimental measurements or other simulation results, the excess adsorption amount should be expressed per unit surface area of clay, rather than per unit mass of clay as is usually used in experiments^8^. The excess adsorption amount per unit surface area of clay is given by


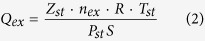


where *Z*_*st*_ is the compress factor of adsorbate molecules under standard state, *R* is the ideal gas constant of 8.314 J/ (mol·K), and *Q*_*ex*_ (cm^3^/m^2^) is the volume of excess adsorption amount per unit clay surface area (*S*, in the unit of m^2^) under the standard state. The temperature (*T*_*st*_) and pressure of standard state are 273.15 K and 0.101 MPa, respectively.

#### Effect of temperature and pressure

**Figure 6:**
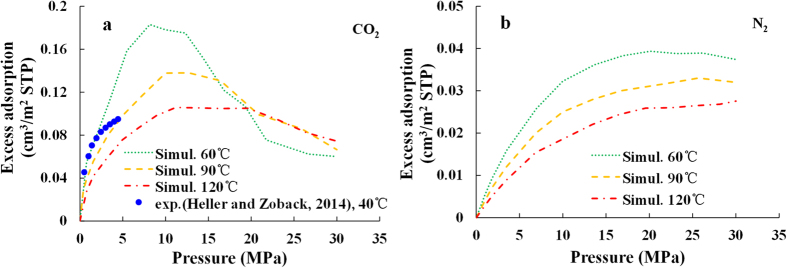
Excess adsorption isotherms of (**a**) CO_2_ and (**b**) N_2_ obtained from simulations at the temperatures of 333, 363 and 393 K (60, 90 and 120 °C) with the pore size of 2 nm and the experimental measurements of CO_2_ adsorption isotherm on natural illite mineral at the temperature of 40 °C reported by Heller and Zoback^41^.

To investigate the temperature effect, the excess adsorption isotherms are compared among different temperatures of 333, 363, and 393 K (60, 90 and 120 °C) for both CO_2_ and N_2_ at the pore size of 2 nm (as shown in Fig. 6). Together with the simulation results, the experimental measurements of CO_2_ adsorption isotherm at 40 °C reported by Heller and Zoback^42^ are also presented in Fig. 6a. The experimental adsorption amounts, originally expressed per unit mass of clay (SCF/ton), are converted to be per unit surface area (cm^3^/m^2^) by normalizing the original data by the specific surface area of illite. The specific surface area of illite with the value of 41 m^2^/g is reported by Macht *et al.*^43^, which is derived by combining the external N_2_ BET specific surface area with the atomic force microscopy specific edge surface area. The simulation results and the experimental measurements are of the same order of magnitude, although they are not matched perfectly. The simulation results are reasonable and acceptable due to the following reasons:

- To compare with the simulation results, the experimental measurements are converted to be expressed per unit surface area by normalizing their original data (expressed per unit mass) by the specific surface area of illite. Therefore, the specific surface area of illite is a key factor that influences the measured excess adsorption amount. However, the measured specific surface area of illite is significantly different from each other according to different experiments, from 2.26 m^2^/g to 62 m^2^/g. As a result, the error in the measured excess adsorption amounts is not negligible.
- When performing the adsorption experiments, the free volume is measured by helium, whose molecular dynamic diameter is smaller than that of CO_2_ and N_2_. Therefore, the experimental adsorption amount obtained using the helium is lower than the theoretical value.
- The adsorption capabilities vary for pores with different pore sizes (see below for further discussion), so we should not expect the simulation results obtained from a specific pore size to match exactly the experimental measurements with complex pore structures.
- The illite mineral used in the adsorption experiment is not pure illite. The existence of other minerals would affect the results of the specific surface area and the adsorption capacity.
- The natural illite mineral is not as ideal as what we used in the simulations. Firstly, the isomorphic replacement should be much more random in both number and position in the natural illite mineral. Secondly, the cations filled in the slit pore space should be more diverse rather than the potassium cations only as used in our model. Thirdly, some lattice imperfection and other cleave surface must also exist in the natural illite mineral, and the adsorption capacity on these surfaces would be different from our simulations^34^.
- The difference in the temperature between the simulations and the experiments would also cause the difference between their results.

As shown in Fig. 6, the excess adsorption amount increases with the increasing pressure to a maximum and then decreases with further increase in the pressure. This can be explained by the fact that while both the adsorption phase density and the expected bulk phase density increase with pressure (as shown in Fig. 6), the adsorption phase density grows faster at low pressure since the adsorption position is surplus, and increases slower at high pressure because the adsorption position is saturated. As shown in Fig. 6a, the higher temperature causes a lower peak and a smaller descending rang in excess adsorption isotherms as the pressure increases beyond the peak. This is due to the exothermic nature of physisorption process. This phenomenon is consistent with the experimental results of the gas adsorption characteristics in shale^6^,^7^.

#### Effect of pore size

**Figure 7:**
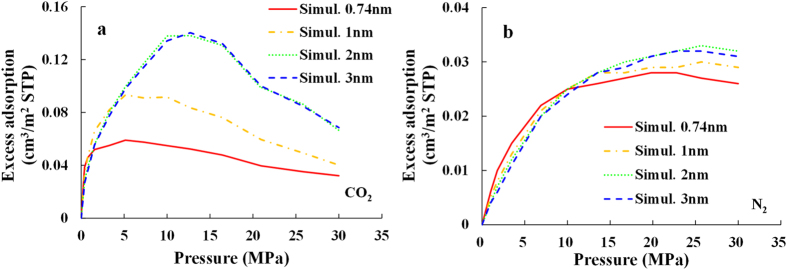
The excess adsorption isotherms of (**a**) CO_2_ and (**b**) N_2_ at the temperature of 363 K (90 °C) for the pore size of 2 nm.

To investigate the effect of the pore size on the excess adsorption amount, the excess adsorption isotherms at the temperature of 90 °C with various pore sizes from 0.74 to 3 nm are calculated and compared in Fig. 7. At low pressure, from 0 to 2 MPa for CO_2_ and from 0 to 10 MPa for N_2_, the adsorption amount decreases with the increasing pore size. This is due to the fact that the strong adsorption layers overlap for the pore size of 0.74 nm and the weak adsorption layers overlap for the pore size of 1 nm, however, no weak adsorption layer occurs for the pore size of 2 nm and 3 nm. In other words, there are more contributors to excess adsorption amount for smaller pore size, this is consistent with the gas density profiles for different pore size presented in Fig. 4. At high pressure, from 2 to 30 MPa for CO_2_ and form 10 to 30 MPa for N_2_, the adsorption amount increases with the increasing pore size. That is due to the fact that the weak adsorption layers occur for the pore size of 2 nm and 3 nm at high pressure. As discussed previously, now that the densities of the strong adsorption layers are similar, the difference in the adsorption amount between different pore sizes (except for 0.74 nm because of the overlap of the strong adsorption layers) mainly depends on the gas densities of the (overlapped) weak adsorption layers and the relative volumes. The volume relative to the weak adsorption layers is larger for the large pore size than that for the small pore size (as shown in Fig. 4), thus the excess adsorption amount increases with the increasing pore size. Generally speaking, the difference in the excess adsorption isotherms between the pore size of 2 nm and 3 nm is negligible, indicating that the overlap of interaction energy and adsorption layers can hardly occur when the pore size is larger than 2 nm.

Meanwhile, more gas amount with the expected bulk gas density will be subtracted from the absolute loading amount to obtain the excess adsorption amount for a larger adsorbed phase volume. For the pore sizes from 0.74 nm to 2 nm, the total pore space is the adsorption phase volume because of the overlap effect of the gas density. As a result, the descending rang of isotherm as the pressure increases beyond the peak increases with the increasing pore size (from 0.74 nm to 2 nm).

The pressure corresponding to the maximum of the excess adsorption isotherms increases with the increasing pore size. This observation was also reported by Tan and Gubbins for methane adsorbed in carbon micro pores^44^ and Liu *et al.* for methane adsorbed in shale^45^. This can be explained by the fact that the sorption potential overlaps more intensely in a smaller pore size, thus the adsorption positions get saturated at lower pressure.

## Conclusions

The GCMC simulations are performed to investigate the adsorption behavior of CO_2_ and N_2_ in K-illite with varying slit pore size from 0.74 nm to 3 nm at different temperatures of 333, 363 and 393 K (60, 90 and 120 °C) over a range of pressures up to 30 MPa. The adsorption mechanisms of CO_2_ and N_2_ in K-illite have been revealed through the investigation of the gas density profile, interaction energy and the excess adsorption amount from the simulation results. The main findings are concluded as follows:

1. The impact of cation dynamics on the adsorption amount of CO_2_ and N_2_ is slight and negligible, therefore, both rigid and dynamic potassium cations in the simulation cells are suitable to investigate the adsorption amount of CO_2_ and N_2_ in K-illite.
2. Although the pore wall thickness of the simulation cell is much thinner than that of natural illite mineral, it will not affect the adsorption behavior as long as it is greater than half layer of clay sheet.
3. It is reasonable to compare the simulation results with the experimental measurements when the excess adsorption amount is expressed in per unit surface area of illite. The consistency between the simulation results and the experimental measurements confirms the validity of the GCMC simulations carried out in this work.
4. The sorption potential overlaps in the micropores and the overlap effect enhances with the decreasing pore size, leading to improving interaction between gas and the clay surface and increasing gas density in the adsorption layers as the pore size drops.

The simulation results of the adsorption amounts, the interaction energies, and the density profiles are all self-consistent. The adsorption mechanisms revealed in this paper improved the understanding of the shale gas adsorption behavior, which could be hopefully expected to be conducive to the shale gas exploration and development in the future.

## Additional Information

**How to cite this article**: Chen, G. *et al.* Research of CO_2_ and N_2_ Adsorption Behavior in K-Illite Slit Pores by GCMC Method. *Sci. Rep.* **6**, 37579; doi: 10.1038/srep37579 (2016).

**Publisher’s note:** Springer Nature remains neutral with regard to jurisdictional claims in published maps and institutional affiliations.

## Figures and Tables

**Table 1:** Kinetic diameters of both CO_2_ and N_2_ molecules and specific surface areas of types I and II K-illite simulation cells.

Adsorbate	Kinetic diameter^12^	Type of simulation cell	Specific surface area (m^2^/g)
CO_2_	0.33	I	1038.92
		II	522.40
N_2_	0.364	I	1015.76
		II	517.04
